# Supplementary material for: Real-World Insights into Efficacy and Safety of Enfortumab Vedotin in Japanese Patients with Metastatic Urothelial Carcinoma: Findings, Considerations, and Future Directions
Source: Curr Oncol. 2024 Jan 29;31(2):759–68. doi: 10.3390/curroncol31020056 (PMC10887831; doi:10.3390/curroncol31020056)
Supplement: Supplementary file 1 [file curroncol-31-00056-s001.zip › curroncol-2706389-supplementary.pdf]

Supplementary Materials

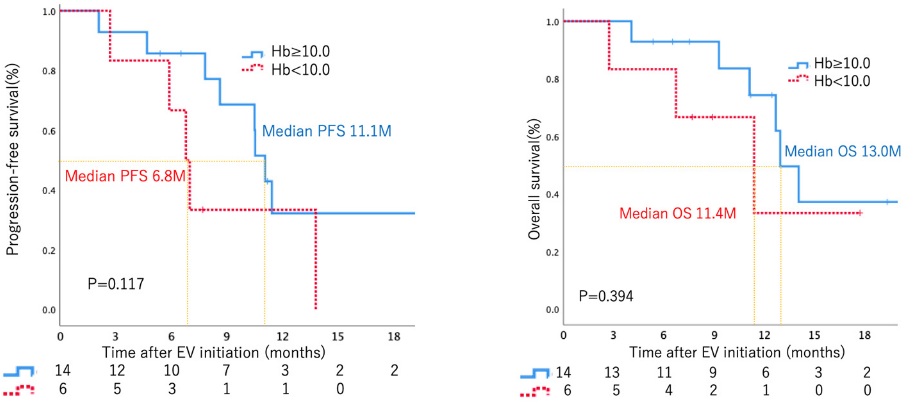

Figure S1. Kaplan-Meier curve comparing progression-free survival (PFS) and overall survival (OS) in patients categorized by the presence or absence of anemia.

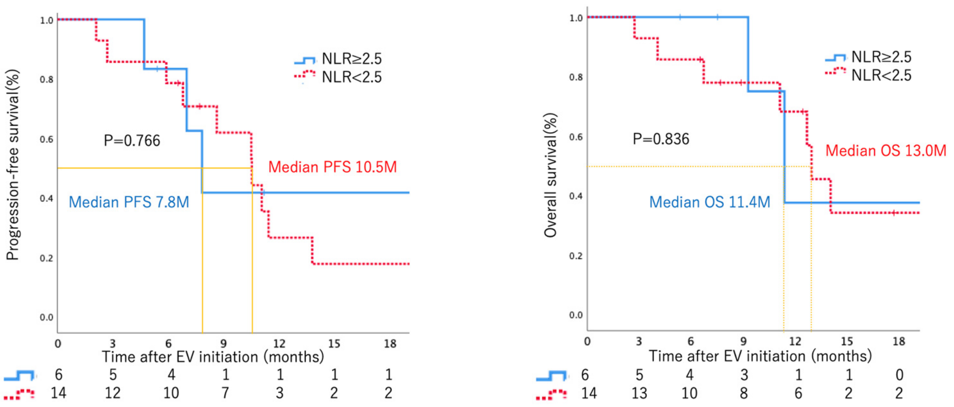

Figure S2. Kaplan-Meier curve comparing progression-free survival (PFS) and overall survival (OS) in patients categorized by a Neutrophil-to-Lymphocyte Ratio (NLR) of more than 2.5 or less.

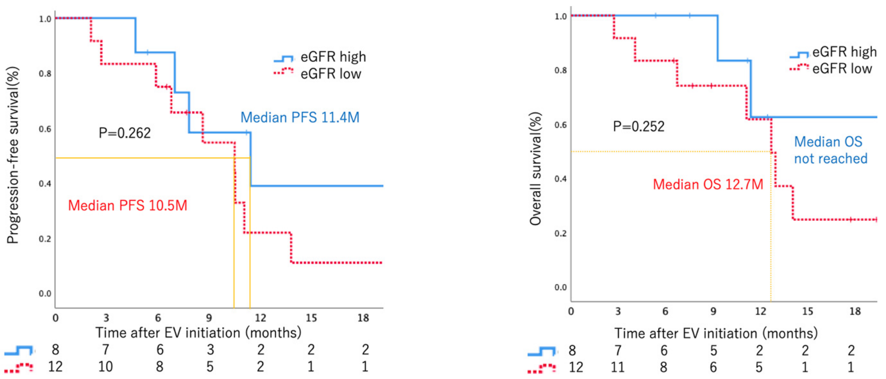

Figure S3. Kaplan-Meier curve comparing progression-free survival (PFS) and overall survival (OS) among patients categorized by a glomerular filtration rate (GFR) exceeding the median

value and those below it.

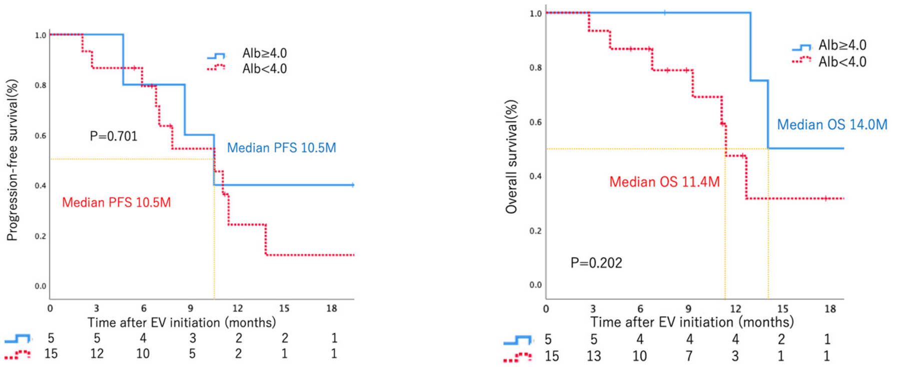

Figure S4. Kaplan-Meier curve comparing progression-free survival (PFS) and overall survival (OS) in patients categorized by a albumin of more than 4.0 mg/dl or less

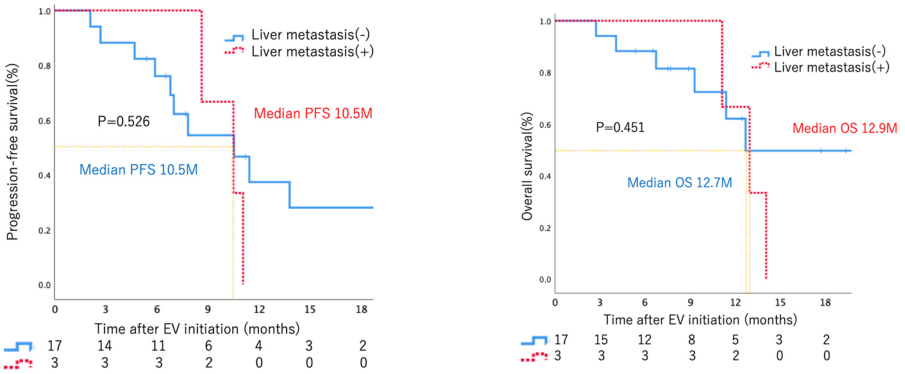

Figure S5. Kaplan-Meier curve comparing progression-free survival (PFS) and overall survival (OS) in patients categorized by the presence or absence of liver metastasis.

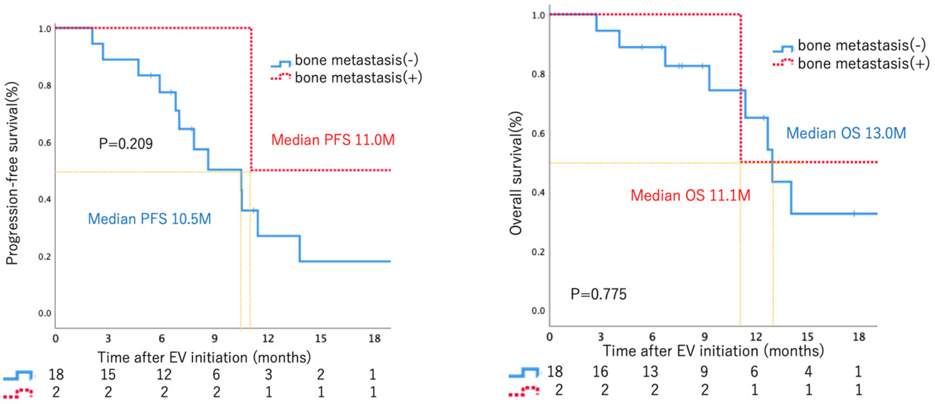

Figure S6. Kaplan-Meier curve comparing progression-free survival (PFS) and overall survival

(OS) in patients categorized by the presence or absence of bone metastasis.

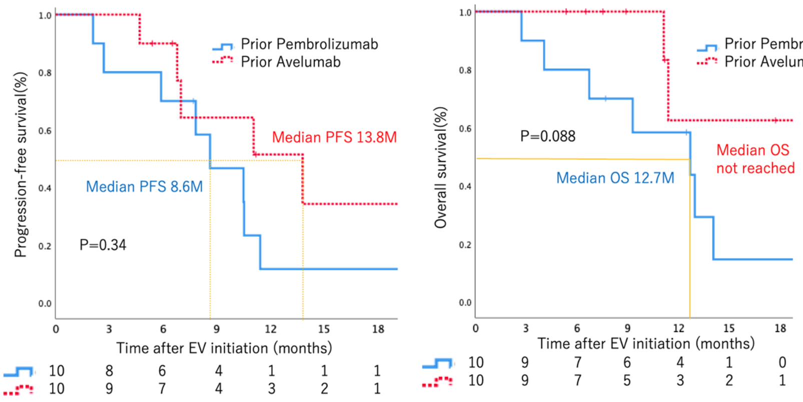

Figure S7. Kaplan-Meier curve comparing progression-free survival (PFS) and overall survival (OS) in patients categorized by the prior immune-checkpoint inhibitors.

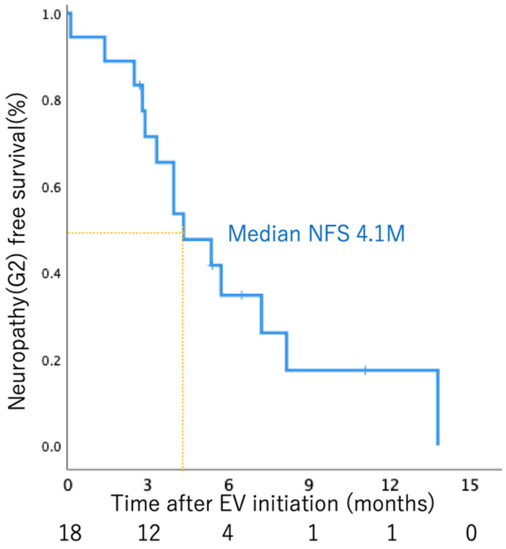

Figure S8. Kaplan–Meier curve of patient G2 neuropathy-free survival rate.

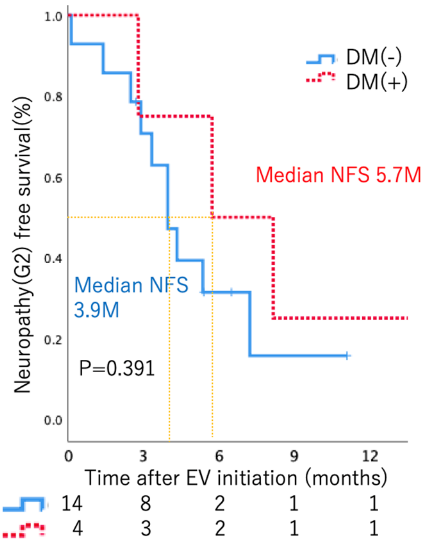

Figure S9. Kaplan–Meier curve comparing the G2 neuropathy free survival rate of patients who had past history of diabetes meritus with who did not.

Table S1. Details of subtypes (n = 8).

Details of subtypes	number
micropapillary	1
lipid-rich	1
sarcomatoid	2
plasmacytoid	1
grandular differentiation	1
micropapillary with lipid-rich	1
micropapillary with squamous differentiation	1
